# The interplay of histone modifications – writers that read

**DOI:** 10.15252/embr.201540945

**Published:** 2015-10-16

**Authors:** Tianyi Zhang, Sarah Cooper, Neil Brockdorff

**Affiliations:** Developmental Epigenetics, Department of Biochemistry, University of OxfordOxford, UK

**Keywords:** chromatin, histone modifications, Polycomb, Trithorax

## Abstract

Histones are subject to a vast array of posttranslational modifications including acetylation, methylation, phosphorylation, and ubiquitylation. The writers of these modifications play important roles in normal development and their mutation or misregulation is linked with both genetic disorders and various cancers. Readers of these marks contain protein domains that allow their recruitment to chromatin. Interestingly, writers often contain domains which can read chromatin marks, allowing the reinforcement of modifications through a positive feedback loop or inhibition of their activity by other modifications. We discuss how such positive reinforcement can result in chromatin states that are robust and can be epigenetically maintained through cell division. We describe the implications of these regulatory systems in relation to modifications including H3K4me3, H3K79me3, and H3K36me3 that are associated with active genes and H3K27me3 and H3K9me3 that have been linked to transcriptional repression. We also review the crosstalk between active and repressive modifications, illustrated by the interplay between the Polycomb and Trithorax histone-modifying proteins, and discuss how this may be important in defining gene expression states during development.

## Introduction

In eukaryotes, DNA is packaged in the form of chromatin. The basic unit of chromatin, the nucleosome, is comprised of 147 bp of DNA wrapped around a histone octamer made of two dimers of H2A and H2B and a tetramer of H3 and H4 proteins. The N- and C-terminal histone tails protrude from the nucleosome core and have the potential to interact with adjacent nucleosomes and the linker DNA. All histones can be posttranslationally modified, and the sites of modification are often on the histone tails. These modifications can regulate chromatin structure directly and frequently act as binding sites for the recruitment of other non-histone proteins to chromatin. The most abundant histone modifications are acetylation, phosphorylation, methylation, and ubiquitylation, although many other modifications have been reported (reviewed recently in 1).

Transcriptionally active and silent chromatin is characterized by distinct posttranslational modifications on the histones or combinations thereof. Active genes typically carry high levels of lysine acetylation on the H3 and H4 tails, trimethylation of H3 lysine 4, trimethylation of H3 lysine 79, ubiquitylation of H2B, and trimethylation of H3 lysine 36 (Fig†). Marks associated with repressed genes include trimethylation of lysine 27, ubiquitylation of H2A on lysine 119, and trimethylation of H3 lysine 9 (Fig†). The chromatin-modifying enzymes that catalyze these marks can be recruited to target sites by sequence-specific DNA-binding transcription factors that regulate transcriptional states of particular genes. However, other more general features of the DNA such as its global CG content and DNA methylation status can be read by the DNA-binding Zn-finger CxxC domain present in many chromatin-modifying enzymes 2. Equally, the act of transcription can direct the recruitment of writers that associate with the transcriptional machinery, leading to the accumulation of specific marks such as H3K4me3 and H3K36me3.

**Figure 1:**
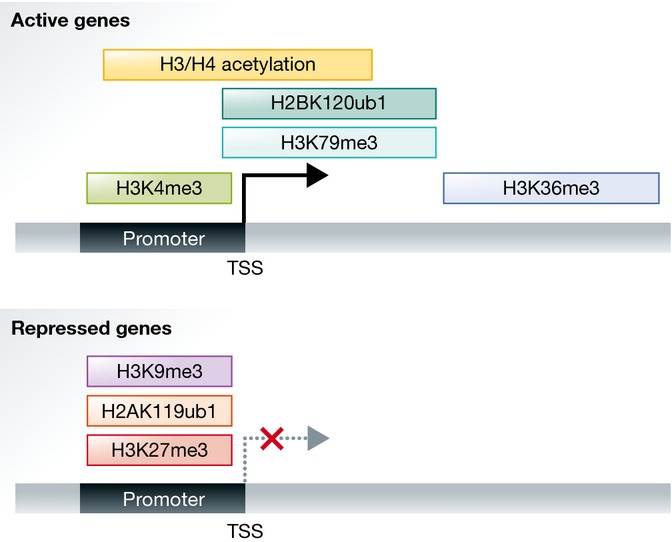
The distribution of histone modifications over active and repressed genes

Given the large number of different histone modifications, the potential combinatorial complexity is vast. Advances in technology over the past decade such as ChIP-sequencing have allowed us to map the distribution and co-localization of histone marks at high-resolution genome wide, while mass spectrometry, often in combination with stable isotope labeling, enables the analysis of histone marks and dynamics at the level of a single histone tail. Interestingly, mass spectrometric data suggest that there are many combinations of modifications that are either more likely to occur together, or are mutually exclusive, suggesting crosstalk between these marks. Such crosstalk can occur in *cis* between distinct modifications on the same histone tail, or in *trans* either on neighboring histones within the same nucleosome or on neighboring nucleosomes in a chromatin domain.

The patterns of histone marks associated with distinct transcriptional states are established through a dynamic interplay between histone readers, writers, and erasers. Importantly, the writers that place these marks contain chromatin-reading domains that can bind preexisting histone marks. Studies have shown that such crosstalk between histone marks can both positively and negatively regulate binding and catalytic activity of writers, resulting in positive and negative feedback loops. Therefore, writers that can also read the histone modifications are required for the establishment and maintenance of chromatin states at active and repressed genes and may play important roles in the memory and switching of gene expression states.

**Figure 2:**
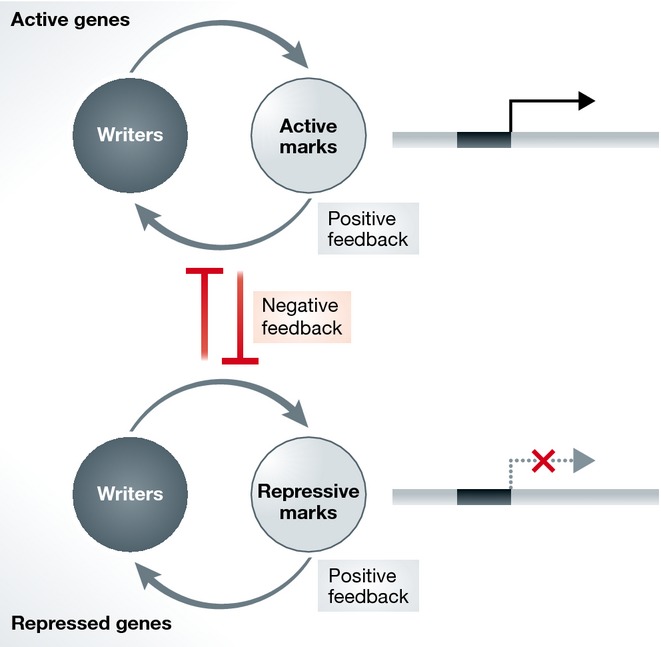
Crosstalk between chromatin writers and histone marks at active and repressed genes Chromatin writers and chromatin marks associated with active genes positively reinforce each other through various positive feedback mechanisms. The same holds true for writers and marks associated with repressed genes. Additionally, negative feedback mechanisms and mutual inhibition between writers and marks associated with the opposite gene expression state also reinforce distinct transcriptional states.

In this review, we will focus on several examples of the positive and negative feedback mechanisms that regulate the formation, reinforcement, and maintenance of the distinct patterns of histone marks associated with active and repressed transcriptional states (Fig2). However, such features are likely to be more general features of chromatin states, and the principles seen in these examples are likely to be applicable to the plethora of other chromatin modifications whose function is still unclear.

## Active histone modifications

In eukaryotic organisms, gene expression is regulated through the synergistic actions of multiple factors, including but not limited to, transcription factors, the transcriptional machinery, chromatin remodelers, and the presence of specific histone variants and histone modifications. Active chromatin domains are characterized by a distinct array of histone marks. H3K27ac and H3K4me1 are associated with active enhancers 3, and high levels of H3K4me3 and H3 and H4 acetylation are found at the promoters of active genes 4–6. The bodies of active genes are enriched in H3 and H4 acetylation 7, H3K79me3 8, and H2BK120u1 9,10, and increasing H3K36me3 toward the 3′ end 11. These histone marks may regulate transcription by creating an open chromatin structure and recruit effectors that mediate a transcriptionally competent state. While the function of many active histone modifications is not fully understood, there is abundant evidence that their deposition is required for the proper regulation of gene expression. Positive crosstalk mechanisms between different histone modifications play an important role in the recruitment and maintenance of active histone modifications at active genes.

### Establishment and maintenance of H3K4me3

**Figure 3:**
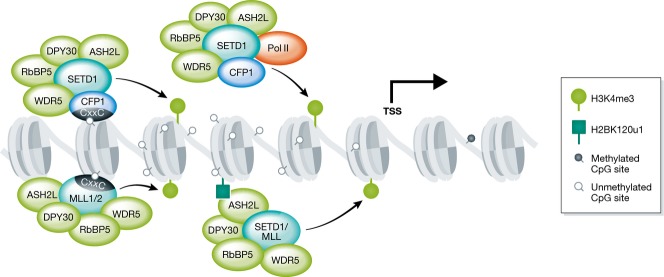
Establishment of H3K4me3 and interplay with H2BK120u1 The SETD1 complex associates with Pol II, and H3K4me3 is deposited co-transcriptionally. CFP1 (associated with SETD1) and MLL1/2 can be recruited to promoters *de novo* via CxxC domain binding to CpG islands. H2BK120u1 can recruit H3K4 writers, possibly through recognition of H2BK120u1 by the ASH2L subunit.

H3K4me3 is a highly conserved histone modification and its association with transcription is evolutionarily conserved in eukaryotes. In mammals, H3K4 methylation is catalyzed by six related homologs of the yeast SET1—SETD1A, SETD1B, MLL1, MLL2, MLL3, and MLL4 12. These complexes are comprised of the catalytic SETD1/MLL subunits and four core subunits WDR5, RBBP5, ASH2L, and DPY30, and as well many other complex-specific subunits 13–15. H3K4me3 is a hallmark of active genes and is distributed along the promoter and TSS regions 6,16,17. Work in yeast shows that SET1 associates with the PAF complex and the Ser5-phosphorylated initiating form of Pol II and is co-transcriptionally deposited 18 (Fig3). Additionally, the recruitment of SETD1 and MLL to specific target genes is mediated by many cell type-specific transcription factors or transcriptional coactivators 19–23. However in higher organisms especially, more general mechanisms of H3K4me3 recruitment and establishment are also at play.

Notably, the distribution of H3K4me3 is highly coupled to the presence of CpG islands, regions of CpG- and GC-dense DNA that are predominately unmethylated and found at 50–70% of vertebrate promoters 24. A biochemical link between CpGI promoters and H3K4me3 was eluciated with the discovery of the Zn-finger CxxC domain which specifically binds nonmethylated CpGs and is present in MLL1/2 and the CFP1 subunit of SETD1A/B 2 (Fig3). All CpGI promoters are marked with H3K4me3, and the level of H3K4me3 is correlated to gene activity 25,26. Emerging evidence suggests that *in vivo*, MLL2 is responsible for maintaining H3K4me3 at CpGI promoters with low expression 27,28, while the SETD1-specific subunit CFP1 is preferentially enriched at active gene promoters with higher levels of H3K4me3 29. In ESCs, CpGI promoters linked to developmentally regulated genes are bivalent and harbor the repressive H3K27me3 mark as well as H3K4me3 30. Importantly, it has been suggested that the ability of H3K4 writers to sample CpGIs genome wide and the presence of H3K4me3 at CpGI promoters may poise silent genes for rapid activation upon differentiation.

SETD1/MLL complexes may reinforce their binding through recognition of their own mark, H3K4me3. The PHD finger domain of CFP1 is known to read H3K4me3 and mediates SETD1 interaction with H3K4me3 31–33. The third PHD domain in MLL1 is important for H3K4me3 binding and MLL1 recruitment to target sites in the Hox locus 34. Other PHD domains within SETD1/MLL may also interact with H3K4me3 but remain to be further characterized 35. The ability of SETD1/MLL to sample promoters and bind H3K4me3 may be involved in the maintenance of this mark at active genes. These mechanisms of H3K4me3 binding by H3K4 writers suggest that once established, this mark may positively reinforce its own deposition.

### Crosstalk between H2BK120u1, H3K4me3, and H3K79me3

One of the best-studied pathways of positive histone crosstalk is the stimulation of H3K4me3 and H3K79me3 by H2BK120u1 (or H2BK123u1 in yeast). In yeast, H2BK123u1 is established by the ubiquitin ligase RAD6/BRE1 during transcriptional initiation and localizes to the TSS and along the bodies of active genes 36. Depletion of RAD6/BRE1 or mutation of H2BK123 causes severe loss of H3K4me3 and H3K79me3 37,38. This positive crosstalk between H2BK123u1 and H3K4me3 and H3K79me3 is specific and does not extend to the regulation of H3K36me3, another mark associated with transcription 37,38.

H2BK123 lies in close proximity to H3K79 on the exposed nucleosome surface, and the H3K79 methyltransferase DOT1 in yeast has been shown to be influenced by deletion and mutation of residues on the H2B tail 39. In humans, the situation is more complex, as H3K79me3 and DOT1L distribution is not solely dependent on H2BK120u1. Human DOT1L localizes at active genes and peaks around the TSS and moreover has been shown to bind both Ser5- and Ser2-phosphorylated forms of the Pol II CTD 40. As such, H3K79me3 is a marker of active genes, yet its exact role in transcriptional regulation remains to be discovered.

The crosstalk between H2BK120u1 and H3K4me3 is conserved in mammals, as knockdown of the BRE1 homologs RNF20/40 leads to global reduction in H3K4me3 41 (Fig3). More recently, studies on the MSL1/MSL2 E3 ligase that catalyzes H2BK34u1 have also revealed a crosstalk between H2BK34u1 and H3K4me3 42. Both H2BK120u1 and H2BK34u1 are now known to allosterically stimulate the activity of the MLL complex through binding to the ASH2L subunit 43. Sites of ubiquitylation at H2BK120 and H2BK34 reside on the nucleosome surface and may provide a more favorable substrate for SET1 or MLL complex binding and activity 43. As ASH2L is a core subunit of all writers of H3K4 methylation, H2B ubiquitylation may be one mechanism of H3K4me3 maintenance at active promoters through a positive feedback loop whereby transcription results in deposition of H2Bub, which subsequently activates the H3K4 methyltransferases.

### H3K4me3 and histone acetylation

Histone lysine acetylation is a highly abundant mark and is known to regulate many cellular processes including transcription. Acetylation of histones H3 and H4 is highly correlated with gene expression. A unique structural motif, the bromodomain, specifically recognizes acetylated lysines and is present in many proteins involved in transcriptional regulation 44. Besides the direct recruitment of effectors, histone acetylation has also been proposed to physically alter chromatin structure by neutralizing the positive charge of lysines and disrupting intra- and internucleosomal interactions, which lead to an open chromatin environment permissible to transcription. Lysine acetylation of three residues on the H3 globular domain H3K56, H3K64, and H3K122, all of which lie at the H3–DNA interface, may disrupt electrostatic interactions within the nucleosome and have been linked to gene activation 45–47. H3K122ac has been shown to directly promote *in vitro* transcription through stimulating histone eviction 47. H3 and H4 histone tail acetylations enhance DNA unwrapping, while H3 acetylation sensitizes nucleosomes to salt-induced dissociation 48.

**Figure 4:**
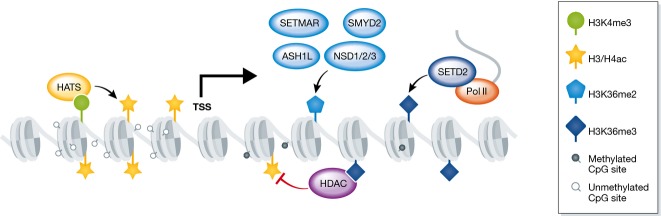
Interplay between H3K4me3, H3K36me3, and H3/H4 acetylation H3K4me3 reinforces H3 and H4 acetylation at the promoters of active genes. Various H3K36 writers catalyze H3K36me1/2 and SETD2 associates with elongating Pol II and catalyzes H3K36me3 co-transcriptionally. H3K36me2/3 recruits HDACs that deacetylate histones over gene bodies.

H3K4me3 and H3/H4 acetylation coexist at the promoter and TSS of active genes, and there are many studies that suggest H3K4me3 promotes downstream H3/H4 acetylation through recruitment of HATs (Fig4). H3K4me3 readers have been identified in many HAT complexes. SGF29, a component of the SAGA HAT complex, contains a tandem tudor domain that binds H3K4me3 and overlaps with H3K4me3 at gene promoters. SGF29 deletion causes loss of H3K9ac and loss of SAGA complex at target sites 49. Similarly, yeast NuA3 50 and NuA4 51, and mammalian HBO1 52 provide other examples of HAT complexes that contain PHD fingers that preferentially bind H3K4me3. Dynamic turnover of H3 lysine acetylation through the combinatorial action of the HAT p300/CBP and HDAC has been shown to occur on histone H3 tails with preexisting H3K4me3, but not other modifications associated with active gene expression such as H3K79me3 or H3K36me3 53. This H3K4me3-linked acetylation is conserved in higher eukaryotes including fly, mouse, and human. Loss of H3K4me3 upon CFP1 deletion leads to loss of CpGI-associated H3K9ac in ESCs 29. Further work using the *Dictyostelium discoideum* model shows that upon knockout of SET1 and loss of H3K4me3, dynamic H3 acetylation was lost 54. The dynamic turnover of acetylation rather than the modification itself may be key in transcriptional activation (reviewed in 55). In support of this, many members of the H3K4me3-binding PHD fingers are associated with HDACs as well as HATs 56. As H3K4me3 has been found to be promoter-associated before transcription initiation, H3K4me3-dependent co-targeting of both HATS and HDACs may facilitate the dynamic turnover of histone acetylation. The above examples illustrate that positive crosstalk between H3K4me3 and histone acetylation is an evolutionarily conserved pathway and that the cooperativity between H3K4me3 and hyperacetylation as well as the dynamic turnover of acetylation is important in ensuring proper transcriptional regulation.

### H3K36me3 and histone deacetylation

Methylation at histone H3K36 is an abundant histone mark highly conserved in eukaryotes. H3K36 mono-, di-, and trimethylation exist in yeast and all of these states are catalyzed by SET2. Mammals on the other hand have multiple writers of H3K36 methylation, including the NSD1/2/3 family, ASH1L, SMYD2, SETMAR, and SETD2, but SETD2 is the sole enzyme responsible for H3K36 trimethylation *in vivo* (reviewed in 57) (Fig4). Interestingly, the uncoupling of H3K36me3 activity from H3K36me1/2 over evolution alludes to specific biologically distinct roles of each methylation state.

H3K36me3 is highly correlated with the transcribed regions of active genes and levels of H3K36me3 increase toward the 3′ end of genes 11. This particular distribution results from the association of Set2 with the elongating Ser2-phosphorylated CTD of Pol II, which is predominant over the bodies and 3′ ends of active genes 58–60. Like H3K4me3, H3K36me3 has also been linked to regulation of histone acetylation. H3K36me3 recruits HDACs to sites of active transcription (Fig4). In yeast, recognition of H3K36me2/3 by the bromodomain-containing EAF3 complex recruits the HDAC RPD3S complex, which deacetylates histones and prevents spurious transcription initiation from within gene bodies 61–63. H3K4me3 and histone hyperacetylation at gene promoters may regulate transcriptional initiation from the TSS, while H3K36me2/3-mediated deacetylation is required in the wake of the transcriptional machinery to prevent initiation from aberrant sites within the gene body. This mutual exclusivity of H3K4me3 and H3K36me3 may be important for maintaining transcriptional integrity. This idea is supported by work showing that promoters lack the H3K36me2/3 mark, and the H3K36me2 demethylases KDM2A/B co-localize with H3K4me3 at CpGI promoters, ensuring active removal of H3K36me2 from transcriptional start sites 64,65.

H3K36me2/3 is recognized by a protein motif, the PWWP domain, found in many nuclear chromatin-binding proteins 66–69. Notably, all three members of the NSD family of H3K36 methyltransferases that catalyze H3K36me1/2 each contain two PWWP domains 70 and have been shown to preferentially bind H3 peptides containing H3K36me3 69. This implies that H3K36me2/3 recognition by its writers may be important for the propagation of H3K36me1 and H3K36me2 at certain sites. Mono-/dimethylation of H3K36 is more pervasive than H3K36me3 and not restricted to sites of active transcription or euchromatin domains 71,72. The biological function of mono-/dimethylation is unknown, though an increase in H3K36me2 levels as a result of mutations in NSD2 has been linked to upregulation of gene expression profiles in cancers 73–75. H3K36me2 may have an important biological function in its own right or may be required to serve as a substrate for subsequent SETD2-mediated H3K36 trimethylation. The broad distribution of H3K36me2 and H3K36me3 over active chromatin may also prevent the spreading and accumulation of silencing marks such as H3K27me3 through direct inhibition of the Polycomb complex PRC2 76,77, which will be discussed below.

## Repressive histone modifications

The methylation of residues lysine 27 and lysine 9 of H3 and the ubiquitinylation of H2A on lysine 119 are hallmarks of repressive chromatin and are often found at silent gene loci. H3K27me3 and H2AK119u1 are associated with the formation of facultative heterochromatin, whereas H3K9me2/3, as well as having important roles in the formation of constitutive heterochromatin, also plays a part in regulating gene expression during development.

### H3K27me3 and H2AK119u1 crosstalk

The Polycomb Repressive Complex 2 (PRC2) is responsible for the methylation of lysine 27 and contains four core subunits, EZH2/1, SUZ12, EED, and RBAP46/8 78. The catalytic subunit is the SET domain-containing protein EZH2 (or the related EZH1), although these enzymes are only functional in the context of the full core complex 79–81. There are also accessory proteins that can associate with the core PRC2 complex to form two types of PRC2: PRC2.1 which includes a Polycomb-like subunit (PCL1/2/3) and PRC2.2 which includes the JARID2 and AEBP2 subunits 82. The function of these accessory proteins remains unclear, although they have been shown to modulate activity of PRC2 and may also play a role in targeting PRC2 to chromatin. PRC2 is able to mono-, di-, and trimethylate H3K27, although there is some dispute if PRC2 is the only H3K27me1 methyltransferase. These different methylation states have very different roles, and although H3K27me3 is linked to gene repression, recent studies have suggested that H3K27me1 may be important for gene activation and is enriched over the bodies of genes 83. The H3K27me2 modification is very prevalent in the genome, with MS/MS analysis demonstrating that it accounts for 60–80% of all nucleosomes in mESCs 84, although little is known about its function or binding proteins. H3K27me3 is the most well-characterized mark in terms of facultative heterochromatin formation and is critical for the repression of key transcriptional regulators during development. Therefore, in terms of gene silencing, we will focus on the trimethylation state of H3K27.

In ES cells, H3K27me3 is present at the promoters of several thousand genes, including the Hox gene clusters, where it is associated with heritable gene silencing 85. H3K27me3 modification is also highly enriched on the inactive X chromosome suggesting a role in facultative heterochromatin formation 86. In more differentiated cell types, larger domains of H3K27me3, termed BLOCS, are often visualized over silent loci in the genome 87. As described above, for many of the enzymes associated with active gene expression, there are also positive feedback loops important for the establishment and spreading of repressive domains. The PRC2 component EED contains an aromatic cage that is able to specifically bind to H3K27me3 88. It has been shown that the binding of PRC2 to the modification it deposits is required for the full establishment of H3K27me3 domains, and such a positive feedback mechanism could also be important for the inheritance of the H3K27me3 mark through cell division 89. PRC2 has also been shown to be stimulated by dense chromatin via an interaction of the SUZ12 subunit with the H3 tail (A31-R42) 90. In this way, positive feedback from the local chromatin structure will also allow robust domains of H3K27me3 to be maintained over repressed genes.

The Polycomb repressive complex 1 (PRC1) is an E3 ubiquitin ligase complex that can modify chromatin by monoubiquitylation of H2A on lysine 119. All PRC1 complexes contain the catalytic RING1A/B subunit, and one of six PCGF proteins 91. The presence of different PCGF subunits is thought to define the class of PRC1 complex, for example, PCGF2 (MEL18) and PCGF4 (BMI) make up the canonical PRC1 complexes which also contain CBX (2,4,6,7,8) and polyhomeotic subunits 92. Variant complexes include either the RYBP or YAF2 protein, the presence of which is mutually exclusive with the CBX component 91,93. These variant complexes, such as the complex containing RING1B/PCGF1/RYBP/BCOR/KDM2B, have been implicated in recruitment of PRC1 and have been shown to have higher H2AK119u1 activity compared with canonical PRC1 complexes 91,94. Interestingly, RYBP also contains a ubiquitin-binding domain and has been shown to bind H2AK119u1 95. This suggests that a positive reinforcement mechanism could be important to establish or maintain high levels of H2AK119u1 at PRC1 target domains, in a similar manner to PRC2 where EED binds to H3K27me3.

Both PRC1 and PRC2, along with their associated chromatin modifications, H2AK119u1 and H3K27me3, have been shown to co-localize at many regions of the genome, such as the promoters of developmentally regulated genes and the inactive X chromosome 96–99. A hierarchical recruitment model, whereby the H3K27me3 modification placed by PRC2 is read by PRC1, has been proposed to explain this co-recruitment of both PRC1 and PRC2 to chromatin 100. This occurs by a specific interaction of the H3K27me3 modification with the chromodomain of the CBX protein found in canonical PRC1 complexes 101. Hence, all PRC2 targets would also become PRC1 targets and a repressive domain would be established. However, this hierarchical model is not able to account for all PRC1 recruitment to chromatin since even in the absence of PRC2, the variant RYBP-containing complexes still localize to the correct regions of the genome 93,102. More recently, data from three laboratories have shown that the reverse mechanism is also possible, whereby PRC1 is recruited first, followed by PRC2. In this model, the H2AK119u1 placed by a variant PRC1 complex is recognized by PRC2 103–105. At present, we do not know how H2AK119u1 is recognized by PRC2, but it has been shown that the PRC2.2 complex (containing the accessory factors AEBP2 and JARID2) is enriched in chromatin containing the H2AK119u1 modification 105. Additionally, *in vitro*, this PRC2.2 complex is more active on an H2AK119u1 nucleosome substrate compared with unmodified nucleosomes 105. A remaining question is whether this PRC2 recruitment to H2AK119u1 is via a direct recruitment mechanism, similar to CBX binding to H3K27me3, or by a change in chromatin state or structure associated with the large H2AK119u1 modification.

**Figure 5:**
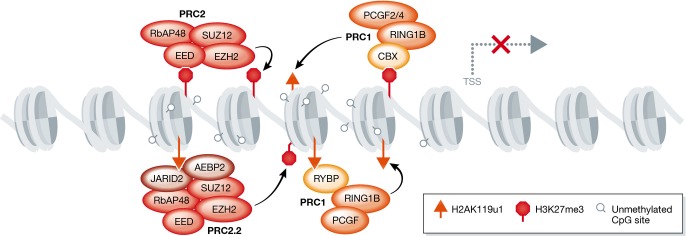
Crosstalk between the Polycomb complexes PRC1 and PRC2 PRC2 reinforces its own mark through binding of EED to H3K27me3. PRC1 may also reinforce its own mark through binding of RYBP to H2AK119u1. Establishment of PRC1 can be reinforced by the presence of PRC2, through recognition of H3K27me3 by the CBX subunit of PRC1. PRC2 establishment can also be reinforced by PRC1 through the recognition of H2AK119u1 by the JARID2/AEBP2 PRC2.2 complex.

In summary, the establishment of Polycomb repressive domains may require these enzymes to read not only their own mark, for example, EED-binding H3K27me3 or RYBP-binding H2AK119u1, but also the marks placed by their partner complex. In this way, H3K27me3 can establish or reinforce H2AK119u1 modifications, and H2AK119u1 can establish or reinforce H3K27me3 deposition (Fig5). Which modification or Polycomb complex is initiating this recruitment is still a matter of debate although recent work has implicated the variant PRC1-KDM2B-containing complex in initiation 106–108. Polycomb target sites overlap with regions of dense unmethylated DNA, CpG islands, and the CxxC domain of KDM2B is able to recognize unmethylated CpGs, providing a plausible mechanism for PRC1 recruitment. Further work is needed to fully understand how both PRC1 and PRC2 complexes are initially recruited to CpG islands. However, once this is established, the positive feedback mechanisms described above involving the histone modifications that these enzymes place will be important to maintain and reinforce their activity at these target sites.

### H3K27me3 and H3K9me2/3 crosstalk

Generally, methylation of H3K9 is associated with constitutive heterochromatin formation and transcriptional silencing. Recently, there has been some evidence that H3K9 methylation can crosstalk with the Polycomb H3K27me3 modification to cooperate in gene repression or as mutually exclusive pathways present at constitutive heterochromatin.

**Figure 6:**
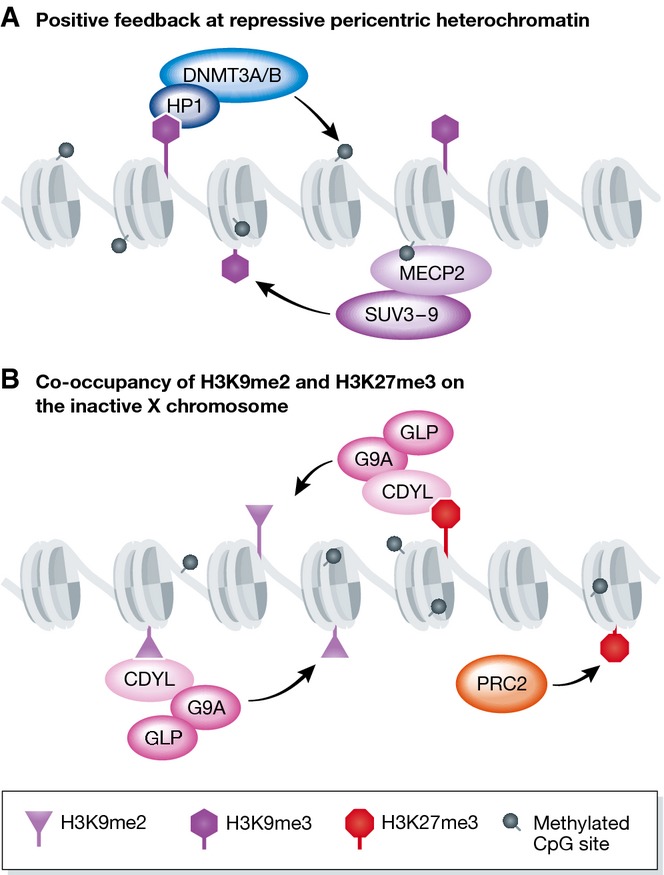
Interplay between H3K9me3, DNA methylation, and H3K27me3 (A) At the pericentric heterochromatin, DNA methylation and H3K9me3 positively reinforce each other. HP1 binds H3K9me3 and recruits the *de novo* DNA methyltransferases DNMT3A/B. MECP2 can bind methylated DNA and recruit the H3K9me3 methyltransferase SUV3-9. (B) H3K27me3 and H3K9me2 coexist on the inactive X chromosome. CDYL may recruit G9a to the inactive X chromosome through its ability to recognize H3K9me2 and H3K27me3. CDYL may reinforce the propagation of H3K9me2 at the Xi.

The heterodimeric complex of G9a and GLP catalyzes H3K9me1 and H3K9me2 modifications 109, which are mainly associated with transcriptional silencing but also occur in euchromatic regions 110. Both proteins contain ankyrin repeat domains that can bind to H3K9me1/2 modifications, allowing the enzymes to read their own marks and therefore allow spreading of the H3K9me2 modification 111. The enzyme SETDB1 can place both H3K9me2/me3 and has roles in repression of transposons, in gene silencing and at pericentric heterochromain 112–115. The SUV3-9H1/H2 enzymes deposit H3K9me2 and H3K9me3 modifications and contain a chromodomain which can recognize these marks 116. A major site of the H3K9me3 modification is at pericentric heterochromatin, where there are self-reinforcing feedback loops involving the chromodomain-containing protein HP1 which can bind to H3K9me3, and recruit *de novo* DNA methyltransferases (DNMT3A/B) 117,118. The resulting DNA methylation can be recognized by MECP2, a protein containing a MBD (methyl binding domain) which can also bind to and recruit SUV3-9 enzymes to pericentric heterochromain 119 (Fig6A). Interestingly at mitosis, H3 is phosophorylated by the kinase Aurora B at H3S10, and this modification next to the H3K9me3 mark causes HP1 to be displaced from the pericentric heterochromatin during this phase of the cell cycle 120.

Several reports have demonstrated that H3K9me3 and H3K27me3 modifications are mutually exclusive 87,103,121,122. In differentiated cells, H3K27me3 BLOCS, which form over silent gene loci, are mutually exclusive with H3K9me3 domains over features such as transposons 87. In SUV3-9H1/H2 knockout cells, there is a loss of H3K9me3 at the pericentric heterochromatin, and a subsequent gain of H3K27me3 103,121, suggesting that not only can these two marks compensate for each other, but that normally H3K9me3 prevents H3K27me3 establishment. A recent study isolating proteins associated with pericentric heterochromatin has shown that a chromatin-associated protein, BEND3, is recruited to pericentric chromatin in the absence of H3K9me3 (or DNA methylation) and is important for recruiting H3K27me3 122. Lack of DNA methylation can also cause H3K27me3 to be recruited to pericentric heterochromatin, but in this case H3K9me3 is still present and forms mutually exclusive domains with H3K27me3, despite both modifications now being enriched at DAPI-dense pericentric regions 103. Recruitment of H3K27me3 to pericentric heterochromatin has also been shown to occur during early mouse development. In the one-cell stage embryo, H3K27me3 can be visualized specifically on the male pericentric heterochromatin, but not the female heterochromatin, which contains H3K9me3 123. In this system, it has recently been shown that it is not the presence of H3K9me3 on the maternal pericentric heterochromatin itself that prevents H3K27me3 recruitment, but rather the presence of HP1b which binds to H3K9me3 124.

Despite reports that H3K27me3 and H3K9me3 are mutually exclusive, a number of ChIP-sequencing studies in ES cells 115, extra-embryonic lineages 125, and differentiated cells 126 have shown that both H3K9me2 and H3K9me3 modifications can coexist with the PRC2 modification H3K27me3 at developmentally repressed genes. Given that both marks are associated with gene repression, it has been suggested that they may cooperate with each other. A mass spectrometry study, in which H3K27me3-containing nucleosomes were purified from HeLa cells, showed that H3K9me2 modifications, and to a lesser extent H3K9me3, were also present with H3K27me3 127. Large-scale proteomic screens have identified several Polycomb proteins as readers of H3K9me3 modifications 31,69. However, the authors suggest that this may be due to the affinity of CBX proteins to H3K27me3 and the high degree of sequence identity surrounding H3K9 and H3K27 (TARKST and AARKSA, respectively). *In vitro* there is no difference in PRC2 activity on nucleosomes that contain H3K9 methylation compared to WT nucleosomes 76. However, a recent paper has found a direct interaction of PRC2 with the G9a/GLP complex and that G9a enzymatic activity (H3K9me2) modulates PRC2 genomic recruitment 128. In addition, studies have reported that PRC2 is necessary for the binding of HP1 to chromatin 129–131. Both H3K27me3 and H3K9me2 modifications have been shown to be present on the inactive X chromosome where the two modifications play complementary roles 132. Here, the molecular mechanisms of crosstalk have been elucidated by the discovery of CDYL, a protein that can bind both H3K9me2 and H3K27me3. CDYL can interact with G9a to propagate the H3K9me2 modification 133. Interestingly, the loss of PRC2, and subsequent loss of H3K27me3, reduces the amount of H3K9me2 present on the Xi, suggesting that CDYL is a link between these two enzymatic activities allowing the combinatorial reading and writing of both modifications (Fig6B).

The mutually exclusive distribution of H3K27me3 and H3K9me3 described above has predominantly been shown in studies of constitutive heterochromatin loci, and in all cases, this clear separation of domains has been seen for trimethylation marks. There are also many examples in which the H3K9me2 modification, and in some cases H3K9me3, can act in concert with H3K27me3, suggesting positive crosstalk between these two mechanisms of heterochromatin formation. In the best-documented examples, this positive crosstalk appears to be between H3K27me3 and H3K9me2. Crosstalk may also vary in different cell types or different differentiation states, in which chromatin structures or the balance of different enzymes may be altered. What is clear is that at least in some circumstances through, for example, CDYL, HP1, or DNA methylation, both the Polycomb system and the H3K9 methylation systems are able to read the chromatin state placed by each other and to write their own modifications accordingly.

As discussed above, both H3K27me3 and H3K9me3 repressive marks can exist as extensive domains within cells. These domains can spread through positive feedback of the writers and their ability to recognize and propagate their marks. Given this, the establishment of boundaries is critical to isolate heterochromatin from euchromatin domains. These boundary elements have been found in multiple eukaryotic organisms, ranging from yeast to human (recently reviewed in 134). Boundaries can be formed by equilibrium between heterochromatin-promoting factors (remodelers, Polycomb proteins, H3K9me3 machinery) and euchromatin-promoting factors (remodelers, transcriptional machinery, Trithorax proteins). Such boundaries could vary in position and this concept forms the basis of position effect variegation (PEV) in which the spread of heterochromatin, for example, domains of H3K9me3, results in the stochastic silencing of a neighboring gene (reviewed in 135). Additionally, *cis*-regulatory elements and the binding of insulator proteins such as CTCF can also determine boundaries. For instance, H3K27me3 domain boundaries within the Hox gene cluster are bound by CTCF; deletion of these CTCF-binding sites results in the infringement of Pol II and H3K4me3 into adjacent heterochromatin territories and disruption of Hox gene silencing 136.

## Interplay between repressive and active chromatin modifications

The main theme that emerges from the data we have described is that chromatin writers, associated with either active or repressed states, are positively regulated by their own marks or marks associated with the same transcriptional state. However, there are clear data that show these chromatin writers can also be negatively influenced by marks associated with the opposite transcriptional state. These negative feedback loops reinforce the maintenance of distinct chromatin states, and may play an important role for switching of gene expression during differentiation and development by creating and reinforcing a bistable state.

Historically, the best-characterized example of an interplay between chromatin complexes in regulating gene expression is the antagonism between Polycomb and Trithorax complexes. *Drosophila* genetics first established Polycomb and Trithorax proteins as two groups having opposing function on Hox gene expression, and subsequently on the regulation of many important developmental genes. Histone crosstalk is important in this interplay, and some of the molecular mechanisms that govern this mutual antagonism between Polycomb and Trithorax proteins and marks have been elucidated.

Schmitges *et al* 76 were the first to show a mechanism of direct inhibition of PRC2 by the TrxG modifications H3K4me2/3 and H3K36me2/3. The catalytic activity of the PRC2 core complex was greatly reduced on recombinant nucleosomes carrying trimethyllysine analogs at H3K4 and H3K36 on the H3 tail. This study and further work demonstrated inhibition of PRC2 activity when the marks H3K4me2/3 and H3K36me2/3 exist in *cis* (on the same histone tail) as the target H3K27. It has been suggested that the allosteric inhibition of the catalytic subunit EZH2 occurs through the recognition of the H3K4me2/3 and H3K36me2/3 marks by the SUZ12 VEFS domain 76. Inhibition of PRC2 by H3K36me2/3 is consistent with mass spectrometry data of the histone H3 peptide fragment K27-R40 isolated from total chromatin from mESCs and transformed cell lines 73,77,137. This shows that trimethylation at H3K27 and H3K36 do not coexist on the same H3 tail or are present at very low levels. Furthermore, removal of SETD2, the only HMT capable of placing H3K36me3, leads to an increase of H3K27me2 over bodies of active genes and reduces levels of expression 83.

It is important to note that although PRC2 is inhibited in *cis* by H3K4me2/3 and H3K36me3, PRC2 is active on nucleosomes harboring these modifications on only one of the two H3 tails, thereby allowing the formation of asymmetrically modified nucleosomes 127. Such nucleosomes have been identified *in vivo* and may represent the nucleosomes present at bivalent promoters in mESCs (promoters that harbor both active and repressive marks, see later).

This negative feedback mechanism has also been shown to operate at a chromosomal level in *C. elegans*. Normally H3K36me3 and H3K27me3 occupy mutually exclusive domains on the autosomes 138. However, removal of the H3K36me1/2 writer MES-4 in the germ line results in a global loss of H3K36me3 leading to the redistribution of H3K27me3 to exogenous sites at germ line-expressed genes, which are normally modified by H3K36me3. This redistribution causes a titration of the H3K27me3 mark from its endogenous sites, including the X chromosomes, and an inability to maintain normal gene expression states 138–140.

As well as the inhibition of Polycomb activity by Trithorax marks, Polycomb marks have also been shown to inhibit the activity of some Trithorax proteins. The best-studied examples are the inhibition of H3K36 methyltransferases by the PRC1 modification H2AK119u1. Yuan *et al* 141 show that the catalytic domain of H3K36 methyltransferases is inhibited by recombinant nucleosomes containing H2AK119u1. Additionally, there is evidence that the PcG PRC1 mark H2AK119u1 inhibits H3K4 methyltransferases MLL1 and possibly MLL3. A study by Endoh *et al* shows that upon RING1A/B knockout and subsequent depletion of H2AK119u1, H3K4me3 levels at several PcG target genes increase 142. Although not extensively investigated, it is possible that H3K27me3 may also inhibit the deposition of H3K4 methylation by the SETD1 and MLL3/4 complexes 143.

These studies provide good evidence that PcG and TrxG marks mutually inhibit the writers associated with the opposing group. It was therefore unexpected when several groups showed that the tudor domains of the PRC2-associated PCL1/2/3 proteins can specifically bind H3K36me3 144–147. Structural and biochemical analyses show that the tudor domain of PCL recognizes H3K36me2/3 with high specificity; however, PCL co-localizes only moderately with H3K36me3 *in vivo* by ChIP-sequencing 144,147. One interpretation of this observation is that the role of this inter-action may be important in the spreading of PRC2 and H3K27me3 to bodies of active genes, and perhaps during the switching of transcriptional state by allowing the initial recruitment of PRC2 to active genes. Consistent with the latter observation, it has been shown that the H3K36 demethylase NO66 can be recruited by PCL3, which would allow for the removal of H3K36me3 before the subsequent acquisition of H3K27me3 146.

H3K27me3 is associated with gene repression, while H3K27ac is associated with gene activation and active enhancers. Since they act on the same lysine residue, these marks are mutually exclusive, and the switch between methylation and acetylation has been well established. The removal of H3K27ac by the NURD complex facilitates the recruitment of PRC2 and accumulation of H3K27me3 at promoters leading to gene repression 148. This process occurs during differentiation of ESCs, when CTBP2 in combination with NURD initiates the silencing of genes that were originally active, through H3K27 deacetylation, allowing deposition of H3K27me3 by PRC2^149^.

Upon loss of H3K9 or DNA methylation, PRC2 accumulates at the pericentric heterochromatin as discussed earlier. In this condition, BEND3 recruits NURD to the pericentric heterochromatin 122, and thus, a similar mechanism of deacetylation of H3K27ac could explain the subsequent PRC2 recruitment and accumulation of H3K27me3 at these sites.

Activation of Polycomb-repressed genes requires a methylation to acetylation switch at H3K27. The phosphorylation of H3S28 on the residue neighboring H3K27 has been shown to mediate this switch. H3S28p inhibits H3K27me3, allowing an accumulation of H3K27 acetylation 150,151. Similarly, the loss of PRC2 subunit SUZ12 and H3K27me3 leads to the accumulation of H3K27ac at PcG target genes 84. It has been suggested that one role of H3K27me3 is to exclude the HATs p300 and CBP, preventing accumulation of H3K27ac at enhancers that is important for gene activation 152. H3K27me2 has been suggested to play a similar role to prevent the firing of non-cell-type-specific enhancers. This idea is supported by the increase of H3K27ac at these enhancers when H3K27 methylation is lost 83.

## Discussion

It is clear that specific histone modifications are associated with the transcriptional state. For many modifications, it is not well established whether they directly influence transcription or their placement is simply a consequence of the transcriptional state present at a particular gene. There are reports that acetylation can directly alter chromatin structure to a more accessible state allowing the recruitment of transcription factors and the transcription machinery. In addition, ubiquitylation of H2A has been shown to inhibit the elongating form of Pol II, suggesting direct effects on the transcriptional state. Conversely, there is substantial evidence to support the idea that transcription factors determine and initiate gene expression, and writers recognize this state and aid in the maintenance of this state through multiple feedback mechanisms. One such mechanism is the recruitment of the H3K4 HMT SETD1 and the H3K36 HMT SETD2 by Pol II itself to deposit histone modifications across the promoter and gene body. The emerging idea that PcG and TrxG can sample CpG islands genome wide and establish domains of repression or activation depending on the transcriptional state at the target promoter also supports the idea that the transcriptional state defines the chromatin modification landscape. This mechanism requires extensive positive and negative crosstalk between these modifications that we have discussed.

Many writers of chromatin modifications are positively regulated by the marks that they place, as well as other marks associated with the same transcriptional state, contributing to the reinforcement of gene expression or silencing. This mechanism could also account for the spreading of marks such as the repressive modifications H3K9me3 and H3K27me3 over large domains in differentiated cells. These reinforcing mechanisms may also play a role in cellular memory by faithful propagation of the histone modifications that allow gene expression profiles to be maintained epigenetically through cell division. There is evidence to suggest that some writers remain associated with chromatin during DNA replication, but the exact molecular mechanisms of this epigenetic memory have yet to be fully elucidated.

Negative histone crosstalk plays an equally important role in dictating distinct chromatin environments. Writers, especially in the case of PcG and TrxG proteins, are often negatively regulated by marks associated with the opposing transcriptional state. As we have seen in the case of H3K27 methylation and acetylation, negative crosstalk is also involved in the switching of gene expression states. It is known that in certain circumstances, marks associated with positive and negative transcription can coexist. In ESCs, bivalent promoters contain both H3K27me3 and H3K4me3, albeit on different histone tails of the same nucleosome, and may represent a chromatin profile amenable to switching between transcriptional states. Genes coding for master transcription factors often have bivalent promoters in ESCs, and their expression is dynamically regulated through development.

The ability of writers to read and place histone modifications is important for the maintenance and regulation of specific transcriptional states throughout development. This is evident by the fact that mice deficient in chromatin-modifying enzymes display severe developmental defects. Several of these enzymes have been known to be involved in various genetic disorders such as Sotos syndrome and Wolf–Hirschhorn syndrome related to translocation of several members of the NSD family of H3K36 methyltransferases 153–155, and cancers related to translocation or mutation of MLL, NSD2, PRC2, SETD2, and countless others 156–158. Most recently, an H3K27M mutation in H3.3 found in pediatric glioma cancer has been shown to deplete levels of H3K27me3 globally potentially through a dominant negative mechanism 159.

In conclusion, the ability of chromatin writers to read preexisting histone modifications contributes in two major ways. First, it allows the maintenance of distinct and robust transcriptional states, which could potentially be propagated through cell division, and therefore act as epigenetically inherited features. Second, crosstalk between modifications and enzymes of opposing transcriptional states can allow the establishment of bistable switches that allow the dynamic regulation of gene expression states. In the future, we hope to understand the extent to which histone crosstalk plays a role in defining the epigenetic landscape of a cell (Sidebar A), and address the role of histone modifications and the crosstalk between them during the processes of development and disease.

**Sidebar A: In need of answers**

- How do chromatin modifications regulate gene transcription? Although there is a clear correlation between chromatin modifications and gene expression states, it will be important to establish the role that modifications play in regulating transcription and indeed whether they are a cause or an effect of transcription.
- What is the role of highly prevalent chromatin modifications such as H3K36me2 or H3K27me2 that mark up to 50% of H3 in mESCs and differentiated tissues 84,121,160? Is it possible that these marks are necessary to reduce noise, for example, by blocking inappropriate histone modifications?
- What is the role of histone variants? H3.3 and H2A.Z are enriched over active genes and may have more specialized regulatory roles compared to their more abundant canonical counterparts 161,162. Histone readers and writers may be sensitive to the histone variant status. For instance, the putative tumor suppressor ZMYND11 is an H3.3 variant-specific reader of H3K36me3 163, while the H3K27 methyltransferase in plants, ATRX5/6, is active on the canonical H3.1 but inhibited by H3.3 164.
- How are epigenetic profiles established within a cell? What are the relative contributions of direct targeting of histone-modifying activities and crosstalk between histone modifications or transcriptional state? For example, are chromatin writers able to sample and read the preexisting chromatin state to determine their activity or binding profiles, or are they directly recruited to their sites of action by sequence-specific DNA-binding factors?
- To what extent are epigenetic modifications maintained through cell division, and do self-reinforcing feedback loops provide a model for the mechanism of such inheritance? Unlike writers and readers, the genomic location of histone modifications can be easily transmitted through both mitosis and meiosis because they are an integral part of the packaging of DNA. However, self-reinforcing loops might become essential after replication to overcome the dilution of old, modified nucleosomes with new nucleosomes and maintain an epigenetic code.
- Do the reciprocal feedback loops between positively and negatively acting histone marks provide the basis for a bistable switch, in which each state is positively reinforced and stable once the initial decision has been made? In order to properly generate and validate such mathematical models, it will be critical to obtain quantitative data on the kinetics and dynamics of the catalytic and binding processes that are involved. This will involve experiments such as FRAP and *in vitro* binding and enzymatic assays, and importantly determining the changes that occur when the system has been perturbed.

## Abbreviations

AEPB2: AE-binding protein 2
ASH2L: absent, small, or homeotic-like
ATRX5/6: *Arabidopsis* Trithorax-related protein 5/6
BEND3: Ben domain containing 3
BLOCS: broad local enrichments
BRE1: Brefeldin-A sensitivity protein 1
CBX: chromobox
CDYL: chromodomain protein, Y-like
CFP1: CXXC finger protein 1
ChIP-sequencing: chromatin immunoprecipitation followed by DNA sequencing
CpG: cytosine-phosphate-guanine
CTCF: CCCTC-binding factor
CTBP2: C-terminal-binding protein 2
CTD: C-terminal domain
DNMT3A/B: DNA methyltransferase 3A/B
DOT1: disruptor of telomeric silencing 1
DOT1L: DOT1-like
DPY30: dumpy-30 protein homolog
EAF3: Esa1p-associated factor 3
EED: embryonic ectoderm development
ESC: embryonic stem cell
EZH2/1: enhancer of zeste homolog 2/1
FRAP: fluorescence recovery after photobleaching
G9a/GLP: G9a and G9a-like protein
H2AK119u1: histone H2A lysine 119 monoubiqutination
H2BK120u1: histone H2B lysine 120 monoubiqutination
H2BK34u1: histone H2B lysine 34 monoubiqutination
H3K27me1/2/3: histone H3 lysine 27 mono/di/trimethylation
H3K36me1/2/3: histone H3 lysine 36 mono/di/trimethylation
H3K4me1/2/3: histone H3 lysine 4 mono/di/trimethylation
H3K9me1/2/3: histone H3 lysine 9 mono/di/trimethylation
HAT: histone acetyltransferase
HBO1: histone acetyltransferase bound to ORC 1
HDAC: histone deactylase complex
HMT: histone methyltransferase
Hox gene: homeobox-containing gene
HP1: heterochromatin protein 1
JARID2: jumonji, AT-rich interactive domain 2
KDM2A/B: lysine demethylase protein 2A/B
MBD: methyl binding domain
MES-4: mesoderm expressed 4
MLL1/2/3/4: mixed-lineage leukemia 1/2/3/4 complex
MSL1/2: male-specific lethal 1/2
NO66: nucleolar protein 66
NSD1/2/3: nuclear receptor-binding SET domain protein 1/2/3
NuA3/4: nucleosomal acetyltransferase of histone H3/H4
NURD: nucleosome remodeling and deacetylase
P300/CBP: P300- and CREB-binding protein
PAF: polymerase-associated factor
PCGF1/2/3/4/5/6: Polycomb group ring finger 1/2/3/4/5/6
PCL1/2/3: Polycomb-like 1/2/3
PH: polyhomeotic
PHD finger: plant homeodomain finger
Pol II: RNA polymerase II
PRC1: Polycomb repressive complex 1
PRC2: Polycomb repressive complex 2
RAD6: ras-related associated with diabetes protein 6
RBBP5: retinoblastoma-binding protein 5
RING1A/B: really interesting new gene 1A/B
RNF20/40: ring finger protein 20/40
RpAb46/48: Rb-associated protein 46/48
RPD3S: reduced potassium dependency 3S complex
RYBP: RING1- and YY1-binding protein
SAGA: Spt-Ada-Gcn5 acetyltransferase
SET domain: Su(var)3-9, enhancer-of-zeste and Trithorax domain
SETD1A/B: SET domain containing 1A/B
SETD2: SET domain containing 2
SETMAR: SET domain and mariner transposase fusion containing protein 2
SMYD2: SET and MYND domain-containing protein 2
SUV3-9 H1/H2: suppressor of variegation 3-9 homolog 1/2
SUZ12: Suppressor of zeste 12 homolog
TrxG: Trithorax group
TSS: transcription start site
WDR5: WD repeat-containing protein 5
YAF2: YY1-associated factor 2
ZMYND11: zinc finger, MYND-type containing 11

## References

[b1] Arnaudo AM, Garcia BA (2013). Proteomic characterization of novel histone post-translational modifications. Epigenetics Chromatin.

[b2] Voo KS, Carlone DL, Jacobsen BM, Flodin A, Skalnik DG (2000). Cloning of a mammalian transcriptional activator that binds unmethylated CpG motifs and shares a CXXC domain with DNA methyltransferase, human trithorax, and methyl-CpG binding domain protein 1. Mol Cell Biol.

[b3] Creyghton MP, Cheng AW, Welstead GG, Kooistra T, Carey BW, Steine EJ, Hanna J, Lodato MA, Frampton GM, Sharp PA (2010). Histone H3K27ac separates active from poised enhancers and predicts developmental state. Proc Natl Acad Sci USA.

[b4] Barrera LO, Li Z, Smith AD, Arden KC, Cavenee WK, Zhang MQ, Green RD, Ren B (2008). Genome-wide mapping and analysis of active promoters in mouse embryonic stem cells and adult organs. Genome Res.

[b5] Deckert J, Struhl K (2001). Histone acetylation at promoters is differentially affected by specific activators and repressors. Mol Cell Biol.

[b6] Liang G, Lin JC, Wei V, Yoo C, Cheng JC, Nguyen CT, Weisenberger DJ, Egger G, Takai D, Gonzales FA (2004). Distinct localization of histone H3 acetylation and H3-K4 methylation to the transcription start sites in the human genome. Proc Natl Acad Sci USA.

[b7] Myers FA, Evans DR, Clayton AL, Thorne AW, Crane-Robinson C (2001). Targeted and extended acetylation of histones H4 and H3 at active and inactive genes in chicken embryo erythrocytes. J Biol Chem.

[b8] Ng HH, Ciccone DN, Morshead KB, Oettinger MA, Struhl K (2003). Lysine-79 of histone H3 is hypomethylated at silenced loci in yeast and mammalian cells: a potential mechanism for position-effect variegation. Proc Natl Acad Sci USA.

[b9] Batta K, Zhang Z, Yen K, Goffman DB, Pugh BF (2011). Genome-wide function of H2B ubiquitylation in promoter and genic regions. Genes Dev.

[b10] Ng HH, Dole S, Struhl K (2003). The Rtf1 component of the Paf1 transcriptional elongation complex is required for ubiquitination of histone H2B. J Biol Chem.

[b11] Pokholok DK, Harbison CT, Levine S, Cole M, Hannett NM, Lee TI, Bell GW, Walker K, Rolfe PA, Herbolsheimer E (2005). Genome-wide map of nucleosome acetylation and methylation in yeast. Cell.

[b12] Sims RJ, Nishioka K, Reinberg D (2003). Histone lysine methylation: a signature for chromatin function. Trends Genet.

[b13] Dehe PM, Dichtl B, Schaft D, Roguev A, Pamblanco M, Lebrun R, Rodriguez-Gil A, Mkandawire M, Landsberg K, Shevchenko A (2006). Protein interactions within the Set1 complex and their roles in the regulation of histone 3 lysine 4 methylation. J Biol Chem.

[b14] Schneider J, Wood A, Lee JS, Schuster R, Dueker J, Maguire C, Swanson SK, Florens L, Washburn MP, Shilatifard A (2005). Molecular regulation of histone H3 trimethylation by COMPASS and the regulation of gene expression. Mol Cell.

[b15] Miller T, Krogan NJ, Dover J, Erdjument-Bromage H, Tempst P, Johnston M, Greenblatt JF, Shilatifard A (2001). COMPASS: a complex of proteins associated with a trithorax-related SET domain protein. Proc Natl Acad Sci USA.

[b16] Mikkelsen TS, Ku M, Jaffe DB, Issac B, Lieberman E, Giannoukos G, Alvarez P, Brockman W, Kim TK, Koche RP (2007). Genome-wide maps of chromatin state in pluripotent and lineage-committed cells. Nature.

[b17] Schneider R, Bannister AJ, Myers FA, Thorne AW, Crane-Robinson C, Kouzarides T (2004). Histone H3 lysine 4 methylation patterns in higher eukaryotic genes. Nat Cell Biol.

[b18] Ng HH, Robert F, Young RA, Struhl K (2003). Targeted recruitment of Set1 histone methylase by elongating Pol II provides a localized mark and memory of recent transcriptional activity. Mol Cell.

[b19] Song ZT, Sun L, Lu SJ, Tian Y, Ding Y, Liu JX (2015). Transcription factor interaction with COMPASS-like complex regulates histone H3K4 trimethylation for specific gene expression in plants. Proc Natl Acad Sci USA.

[b20] Katada S, Sassone-Corsi P (2010). The histone methyltransferase MLL1 permits the oscillation of circadian gene expression. Nat Struct Mol Biol.

[b21] Okuda H, Kawaguchi M, Kanai A, Matsui H, Kawamura T, Inaba T, Kitabayashi I, Yokoyama A (2014). MLL fusion proteins link transcriptional coactivators to previously active CpG-rich promoters. Nucleic Acids Res.

[b22] Narayanan A, Ruyechan WT, Kristie TM (2007). The coactivator host cell factor-1 mediates Set1 and MLL1 H3K4 trimethylation at herpesvirus immediate early promoters for initiation of infection. Proc Natl Acad Sci USA.

[b23] Yokoyama A, Wang Z, Wysocka J, Sanyal M, Aufiero DJ, Kitabayashi I, Herr W, Cleary ML (2004). Leukemia proto-oncoprotein MLL forms a SET1-like histone methyltransferase complex with menin to regulate Hox gene expression. Mol Cell Biol.

[b24] Deaton AM, Bird A (2011). CpG islands and the regulation of transcription. Genes Dev.

[b25] Barski A, Cuddapah S, Cui K, Roh TY, Schones DE, Wang Z, Wei G, Chepelev I, Zhao K (2007). High-resolution profiling of histone methylations in the human genome. Cell.

[b26] Guenther MG, Levine SS, Boyer LA, Jaenisch R, Young RA (2007). A chromatin landmark and transcription initiation at most promoters in human cells. Cell.

[b27] Hu D, Garruss AS, Gao X, Morgan MA, Cook M, Smith ER, Shilatifard A (2013). The Mll2 branch of the COMPASS family regulates bivalent promoters in mouse embryonic stem cells. Nat Struct Mol Biol.

[b28] Denissov S, Hofemeister H, Marks H, Kranz A, Ciotta G, Singh S, Anastassiadis K, Stunnenberg HG, Stewart AF (2014). Mll2 is required for H3K4 trimethylation on bivalent promoters in embryonic stem cells, whereas Mll1 is redundant. Development.

[b29] Clouaire T, Webb S, Skene P, Illingworth R, Kerr A, Andrews R, Lee JH, Skalnik D, Bird A (2012). Cfp1 integrates both CpG content and gene activity for accurate H3K4me3 deposition in embryonic stem cells. Genes Dev.

[b30] Bernstein BE, Mikkelsen TS, Xie X, Kamal M, Huebert DJ, Cuff J, Fry B, Meissner A, Wernig M, Plath K (2006). A bivalent chromatin structure marks key developmental genes in embryonic stem cells. Cell.

[b31] Eberl HC, Spruijt CG, Kelstrup CD, Vermeulen M, Mann M (2013). A map of general and specialized chromatin readers in mouse tissues generated by label-free interaction proteomics. Mol Cell.

[b32] Shi X, Kachirskaia I, Walter KL, Kuo JH, Lake A, Davrazou F, Chan SM, Martin DG, Fingerman IM, Briggs SD (2007). Proteome-wide analysis in Saccharomyces cerevisiae identifies several PHD fingers as novel direct and selective binding modules of histone H3 methylated at either lysine 4 or lysine 36. J Biol Chem.

[b33] Murton BL, Chin WL, Ponting CP, Itzhaki LS (2010). Characterising the binding specificities of the subunits associated with the KMT2/Set1 histone lysine methyltransferase. J Mol Biol.

[b34] Wang Z, Song J, Milne TA, Wang GG, Li H, Allis CD, Patel DJ (2010). Pro isomerization in MLL1 PHD3-bromo cassette connects H3K4me readout to CyP33 and HDAC-mediated repression. Cell.

[b35] Ali M, Hom RA, Blakeslee W, Ikenouye L, Kutateladze TG (2014). Diverse functions of PHD fingers of the MLL/KMT2 subfamily. Biochim Biophys Acta.

[b36] Schulze JM, Hentrich T, Nakanishi S, Gupta A, Emberly E, Shilatifard A, Kobor MS (2011). Splitting the task: Ubp8 and Ubp10 deubiquitinate different cellular pools of H2BK123. Genes Dev.

[b37] Sun ZW, Allis CD (2002). Ubiquitination of histone H2B regulates H3 methylation and gene silencing in yeast. Nature.

[b38] Ng HH, Xu RM, Zhang Y, Struhl K (2002). Ubiquitination of histone H2B by Rad6 is required for efficient Dot1-mediated methylation of histone H3 lysine 79. J Biol Chem.

[b39] Guan X, Rastogi N, Parthun MR, Freitas MA (2013). Discovery of histone modification crosstalk networks by stable isotope labeling of amino acids in cell culture mass spectrometry (SILAC MS). Mol Cell Proteomics.

[b40] Kim SK, Jung I, Lee H, Kang K, Kim M, Jeong K, Kwon CS, Han YM, Kim YS, Kim D (2012). Human histone H3K79 methyltransferase DOT1L protein [corrected] binds actively transcribing RNA polymerase II to regulate gene expression. J Biol Chem.

[b41] Kim J, Hake SB, Roeder RG (2005). The human homolog of yeast BRE1 functions as a transcriptional coactivator through direct activator interactions. Mol Cell.

[b42] Wu L, Zee BM, Wang Y, Garcia BA, Dou Y (2011). The RING finger protein MSL2 in the MOF complex is an E3 ubiquitin ligase for H2B K34 and is involved in crosstalk with H3K4 and K79 methylation. Mol Cell.

[b43] Wu L, Lee SY, Zhou B, Nguyen UT, Muir TW, Tan S, Dou Y (2013). ASH2L regulates ubiquitylation signaling to MLL: trans-regulation of H3K4 methylation in higher eukaryotes. Mol Cell.

[b44] Zeng L, Zhou MM (2002). Bromodomain: an acetyl-lysine binding domain. FEBS Lett.

[b45] Yuan J, Pu M, Zhang Z, Lou Z (2009). Histone H3-K56 acetylation is important for genomic stability in mammals. Cell Cycle.

[b46] Di Cerbo V, Mohn F, Ryan DP, Montellier E, Kacem S, Tropberger P, Kallis E, Holzner M, Hoerner L, Feldmann A (2014). Acetylation of histone H3 at lysine 64 regulates nucleosome dynamics and facilitates transcription. elife.

[b47] Tropberger P, Pott S, Keller C, Kamieniarz-Gdula K, Caron M, Richter F, Li G, Mittler G, Liu ET, Buhler M (2013). Regulation of transcription through acetylation of H3K122 on the lateral surface of the histone octamer. Cell.

[b48] Gansen A, Toth K, Schwarz N, Langowski J (2015). Opposing roles of H3- and H4-acetylation in the regulation of nucleosome structure–a FRET study. Nucleic Acids Res.

[b49] Bian C, Xu C, Ruan J, Lee KK, Burke TL, Tempel W, Barsyte D, Li J, Wu M, Zhou BO (2011). Sgf29 binds histone H3K4me2/3 and is required for SAGA complex recruitment and histone H3 acetylation. EMBO J.

[b50] Taverna SD, Ilin S, Rogers RS, Tanny JC, Lavender H, Li H, Baker L, Boyle J, Blair LP, Chait BT (2006). Yng1 PHD finger binding to H3 trimethylated at K4 promotes NuA3 HAT activity at K14 of H3 and transcription at a subset of targeted ORFs. Mol Cell.

[b51] Doyon Y, Selleck W, Lane WS, Tan S, Cote J (2004). Structural and functional conservation of the NuA4 histone acetyltransferase complex from yeast to humans. Mol Cell Biol.

[b52] Hung T, Binda O, Champagne KS, Kuo AJ, Johnson K, Chang HY, Simon MD, Kutateladze TG, Gozani O (2009). ING4 mediates crosstalk between histone H3K4 trimethylation and H3 acetylation to attenuate cellular transformation. Mol Cell.

[b53] Crump NT, Hazzalin CA, Bowers EM, Alani RM, Cole PA, Mahadevan LC (2011). Dynamic acetylation of all lysine-4 trimethylated histone H3 is evolutionarily conserved and mediated by p300/CBP. Proc Natl Acad Sci USA.

[b54] Hsu DW, Chubb JR, Muramoto T, Pears CJ, Mahadevan LC (2012). Dynamic acetylation of lysine-4-trimethylated histone H3 and H3 variant biology in a simple multicellular eukaryote. Nucleic Acids Res.

[b55] Clayton AL, Hazzalin CA, Mahadevan LC (2006). Enhanced histone acetylation and transcription: a dynamic perspective. Mol Cell.

[b56] Doyon Y, Cayrou C, Ullah M, Landry AJ, Cote V, Selleck W, Lane WS, Tan S, Yang XJ, Cote J (2006). ING tumor suppressor proteins are critical regulators of chromatin acetylation required for genome expression and perpetuation. Mol Cell.

[b57] Wagner EJ, Carpenter PB (2012). Understanding the language of Lys36 methylation at histone H3. Nat Rev Mol Cell Biol.

[b58] Li B, Howe L, Anderson S, Yates JR, Workman JL (2003). The Set2 histone methyltransferase functions through the phosphorylated carboxyl-terminal domain of RNA polymerase II. J Biol Chem.

[b59] Kizer KO, Phatnani HP, Shibata Y, Hall H, Greenleaf AL, Strahl BD (2005). A novel domain in Set2 mediates RNA polymerase II interaction and couples histone H3K36 methylation with transcript elongation. Mol Cell Biol.

[b60] Morris SA, Shibata Y, Noma K, Tsukamoto Y, Warren E, Temple B, Grewal SI, Strahl BD (2005). Histone H3K36 methylation is associated with transcription elongation in Schizosaccharomyces pombe. Eukaryot Cell.

[b61] Joshi AA, Struhl K (2005). Eaf3 chromodomain interaction with methylated H3-K36 links histone deacetylation to Pol II elongation. Mol Cell.

[b62] Carrozza MJ, Li B, Florens L, Suganuma T, Swanson SK, Lee KK, Shia WJ, Anderson S, Yates J, Washburn MP (2005). Histone H3 methylation by Set2 directs deacetylation of coding regions by Rpd3S to suppress spurious intragenic transcription. Cell.

[b63] Keogh MC, Kurdistani SK, Morris SA, Ahn SH, Podolny V, Collins SR, Schuldiner M, Chin K, Punna T, Thompson NJ (2005). Cotranscriptional set2 methylation of histone H3 lysine 36 recruits a repressive Rpd3 complex. Cell.

[b64] Blackledge NP, Zhou JC, Tolstorukov MY, Farcas AM, Park PJ, Klose RJ (2010). CpG islands recruit a histone H3 lysine 36 demethylase. Mol Cell.

[b65] He J, Kallin EM, Tsukada Y, Zhang Y (2008). The H3K36 demethylase Jhdm1b/Kdm2b regulates cell proliferation and senescence through p15(Ink4b). Nat Struct Mol Biol.

[b66] Dhayalan A, Rajavelu A, Rathert P, Tamas R, Jurkowska RZ, Ragozin S, Jeltsch A (2010). The Dnmt3a PWWP domain reads histone 3 lysine 36 trimethylation and guides DNA methylation. J Biol Chem.

[b67] van Nuland R, van Schaik FM, Simonis M, van Heesch S, Cuppen E, Boelens R, Timmers HM, van Ingen H (2013). Nucleosomal DNA binding drives the recognition of H3K36-methylated nucleosomes by the PSIP1-PWWP domain. Epigenetics Chromatin.

[b68] Maltby VE, Martin BJ, Schulze JM, Johnson I, Hentrich T, Sharma A, Kobor MS, Howe L (2012). Histone H3 lysine 36 methylation targets the Isw1b remodeling complex to chromatin. Mol Cell Biol.

[b69] Vermeulen M, Eberl HC, Matarese F, Marks H, Denissov S, Butter F, Lee KK, Olsen JV, Hyman AA, Stunnenberg HG (2010). Quantitative interaction proteomics and genome-wide profiling of epigenetic histone marks and their readers. Cell.

[b70] Stec I, Nagl SB, van Ommen GJ, den Dunnen JT (2000). The PWWP domain: a potential protein-protein interaction domain in nuclear proteins influencing differentiation?. FEBS Lett.

[b71] Kim A, Kiefer CM, Dean A (2007). Distinctive signatures of histone methylation in transcribed coding and noncoding human beta-globin sequences. Mol Cell Biol.

[b72] Schneider TD, Arteaga-Salas JM, Mentele E, David R, Nicetto D, Imhof A, Rupp RA (2011). Stage-specific histone modification profiles reveal global transitions in the Xenopus embryonic epigenome. PLoS ONE.

[b73] Zheng Y, Sweet SM, Popovic R, Martinez-Garcia E, Tipton JD, Thomas PM, Licht JD, Kelleher NL (2012). Total kinetic analysis reveals how combinatorial methylation patterns are established on lysines 27 and 36 of histone H3. Proc Natl Acad Sci USA.

[b74] Martinez-Garcia E, Popovic R, Min DJ, Sweet SM, Thomas PM, Zamdborg L, Heffner A, Will C, Lamy L, Staudt LM (2011). The MMSET histone methyl transferase switches global histone methylation and alters gene expression in t(4;14) multiple myeloma cells. Blood.

[b75] Jaffe JD, Wang Y, Chan HM, Zhang J, Huether R, Kryukov GV, Bhang HE, Taylor JE, Hu M, Englund NP (2013). Global chromatin profiling reveals NSD2 mutations in pediatric acute lymphoblastic leukemia. Nat Genet.

[b76] Schmitges FW, Prusty AB, Faty M, Stutzer A, Lingaraju GM, Aiwazian J, Sack R, Hess D, Li L, Zhou S (2011). Histone methylation by PRC2 is inhibited by active chromatin marks. Mol Cell.

[b77] Yuan W, Xu M, Huang C, Liu N, Chen S, Zhu B (2011). H3K36 methylation antagonizes PRC2-mediated H3K27 methylation. J Biol Chem.

[b78] Kuzmichev A, Nishioka K, Erdjument-Bromage H, Tempst P, Reinberg D (2002). Histone methyltransferase activity associated with a human multiprotein complex containing the Enhancer of Zeste protein. Genes Dev.

[b79] Cao R, Zhang Y (2004). SUZ12 is required for both the histone methyltransferase activity and the silencing function of the EED-EZH2 complex. Mol Cell.

[b80] Nekrasov M, Wild B, Muller J (2005). Nucleosome binding and histone methyltransferase activity of Drosophila PRC2. EMBO Rep.

[b81] Han Z, Xing X, Hu M, Zhang Y, Liu P, Chai J (2007). Structural basis of EZH2 recognition by EED. Structure.

[b82] Alekseyenko AA, Gorchakov AA, Kharchenko PV, Kuroda MI (2014). Reciprocal interactions of human C10orf12 and C17orf96 with PRC2 revealed by BioTAP-XL cross-linking and affinity purification. Proc Natl Acad Sci USA.

[b83] Ferrari KJ, Scelfo A, Jammula S, Cuomo A, Barozzi I, Stutzer A, Fischle W, Bonaldi T, Pasini D (2014). Polycomb-dependent H3K27me1 and H3K27me2 regulate active transcription and enhancer fidelity. Mol Cell.

[b84] Jung HR, Pasini D, Helin K, Jensen ON (2010). Quantitative mass spectrometry of histones H3.2 and H3.3 in Suz12-deficient mouse embryonic stem cells reveals distinct, dynamic post-translational modifications at Lys-27 and Lys-36. Mol Cell Proteomics.

[b85] Bracken AP, Dietrich N, Pasini D, Hansen KH, Helin K (2006). Genome-wide mapping of Polycomb target genes unravels their roles in cell fate transitions. Genes Dev.

[b86] Silva J, Mak W, Zvetkova I, Appanah R, Nesterova TB, Webster Z, Peters AH, Jenuwein T, Otte AP, Brockdorff N (2003). Establishment of histone h3 methylation on the inactive X chromosome requires transient recruitment of Eed-Enx1 polycomb group complexes. Dev Cell.

[b87] Pauler FM, Sloane MA, Huang R, Regha K, Koerner MV, Tamir I, Sommer A, Aszodi A, Jenuwein T, Barlow DP (2009). H3K27me3 forms BLOCs over silent genes and intergenic regions and specifies a histone banding pattern on a mouse autosomal chromosome. Genome Res.

[b88] Margueron R, Justin N, Ohno K, Sharpe ML, Son J, Drury WJ, Voigt P, Martin SR, Taylor WR, De Marco V (2009). Role of the polycomb protein EED in the propagation of repressive histone marks. Nature.

[b89] Hansen KH, Bracken AP, Pasini D, Dietrich N, Gehani SS, Monrad A, Rappsilber J, Lerdrup M, Helin K (2008). A model for transmission of the H3K27me3 epigenetic mark. Nat Cell Biol.

[b90] Yuan W, Wu T, Fu H, Dai C, Wu H, Liu N, Li X, Xu M, Zhang Z, Niu T (2012). Dense chromatin activates Polycomb repressive complex 2 to regulate H3 lysine 27 methylation. Science.

[b91] Gao Z, Zhang J, Bonasio R, Strino F, Sawai A, Parisi F, Kluger Y, Reinberg D (2012). PCGF homologs, CBX proteins, and RYBP define functionally distinct PRC1 family complexes. Mol Cell.

[b92] Morey L, Pascual G, Cozzuto L, Roma G, Wutz A, Benitah SA, Di Croce L (2012). Nonoverlapping functions of the Polycomb group Cbx family of proteins in embryonic stem cells. Cell Stem Cell.

[b93] Tavares L, Dimitrova E, Oxley D, Webster J, Poot R, Demmers J, Bezstarosti K, Taylor S, Ura H, Koide H (2012). RYBP-PRC1 complexes mediate H2A ubiquitylation at polycomb target sites independently of PRC2 and H3K27me3. Cell.

[b94] Morey L, Aloia L, Cozzuto L, Benitah SA, Di Croce L (2013). RYBP and Cbx7 define specific biological functions of polycomb complexes in mouse embryonic stem cells. Cell Rep.

[b95] Arrigoni R, Alam SL, Wamstad JA, Bardwell VJ, Sundquist WI, Schreiber-Agus N (2006). The Polycomb-associated protein Rybp is a ubiquitin binding protein. FEBS Lett.

[b96] Ku M, Koche RP, Rheinbay E, Mendenhall EM, Endoh M, Mikkelsen TS, Presser A, Nusbaum C, Xie X, Chi AS (2008). Genomewide analysis of PRC1 and PRC2 occupancy identifies two classes of bivalent domains. PLoS Genet.

[b97] Plath K, Fang J, Mlynarczyk-Evans SK, Cao R, Worringer KA, Wang H, de la Cruz CC, Otte AP, Panning B, Zhang Y (2003). Role of histone H3 lysine 27 methylation in X inactivation. Science.

[b98] Mak W, Baxter J, Silva J, Newall AE, Otte AP, Brockdorff N (2002). Mitotically stable association of polycomb group proteins eed and enx1 with the inactive x chromosome in trophoblast stem cells. Curr Biol.

[b99] de Napoles M, Mermoud JE, Wakao R, Tang YA, Endoh M, Appanah R, Nesterova TB, Silva J, Otte AP, Vidal M (2004). Polycomb group proteins Ring1A/B link ubiquitylation of histone H2A to heritable gene silencing and X inactivation. Dev Cell.

[b100] Wang L, Brown JL, Cao R, Zhang Y, Kassis JA, Jones RS (2004). Hierarchical recruitment of polycomb group silencing complexes. Mol Cell.

[b101] Ren X, Vincenz C, Kerppola TK (2008). Changes in the distributions and dynamics of polycomb repressive complexes during embryonic stem cell differentiation. Mol Cell Biol.

[b102] Schoeftner S, Sengupta AK, Kubicek S, Mechtler K, Spahn L, Koseki H, Jenuwein T, Wutz A (2006). Recruitment of PRC1 function at the initiation of X inactivation independent of PRC2 and silencing. EMBO J.

[b103] Cooper S, Dienstbier M, Hassan R, Schermelleh L, Sharif J, Blackledge NP, De Marco V, Elderkin S, Koseki H, Klose R (2014). Targeting polycomb to pericentric heterochromatin in embryonic stem cells reveals a role for H2AK119u1 in PRC2 recruitment. Cell Rep.

[b104] Blackledge NP, Farcas AM, Kondo T, King HW, McGouran JF, Hanssen LL, Ito S, Cooper S, Kondo K, Koseki Y (2014). Variant PRC1 complex-dependent H2A ubiquitylation drives PRC2 recruitment and polycomb domain formation. Cell.

[b105] Kalb R, Latwiel S, Baymaz HI, Jansen PW, Muller CW, Vermeulen M, Muller J (2014). Histone H2A monoubiquitination promotes histone H3 methylation in Polycomb repression. Nat Struct Mol Biol.

[b106] Farcas AM, Blackledge NP, Sudbery I, Long HK, McGouran JF, Rose NR, Lee S, Sims D, Cerase A, Sheahan TW (2012). KDM2B links the Polycomb Repressive Complex 1 (PRC1) to recognition of CpG islands. elife.

[b107] Wu X, Johansen JV, Helin K (2013). Fbxl10/Kdm2b recruits polycomb repressive complex 1 to CpG islands and regulates H2A ubiquitylation. Mol Cell.

[b108] He J, Shen L, Wan M, Taranova O, Wu H, Zhang Y (2013). Kdm2b maintains murine embryonic stem cell status by recruiting PRC1 complex to CpG islands of developmental genes. Nat Cell Biol.

[b109] Shinkai Y, Tachibana M (2011). H3K9 methyltransferase G9a and the related molecule GLP. Genes Dev.

[b110] Tachibana M, Ueda J, Fukuda M, Takeda N, Ohta T, Iwanari H, Sakihama T, Kodama T, Hamakubo T, Shinkai Y (2005). Histone methyltransferases G9a and GLP form heteromeric complexes and are both crucial for methylation of euchromatin at H3-K9. Genes Dev.

[b111] Collins RE, Northrop JP, Horton JR, Lee DY, Zhang X, Stallcup MR, Cheng X (2008). The ankyrin repeats of G9a and GLP histone methyltransferases are mono- and dimethyllysine binding modules. Nat Struct Mol Biol.

[b112] Karimi MM, Goyal P, Maksakova IA, Bilenky M, Leung D, Tang JX, Shinkai Y, Mager DL, Jones S, Hirst M (2011). DNA methylation and SETDB1/H3K9me3 regulate predominantly distinct sets of genes, retroelements, and chimeric transcripts in mESCs. Cell Stem Cell.

[b113] Wang H, An W, Cao R, Xia L, Erdjument-Bromage H, Chatton B, Tempst P, Roeder RG, Zhang Y (2003). mAM facilitates conversion by ESET of dimethyl to trimethyl lysine 9 of histone H3 to cause transcriptional repression. Mol Cell.

[b114] Dodge JE, Kang YK, Beppu H, Lei H, Li E (2004). Histone H3-K9 methyltransferase ESET is essential for early development. Mol Cell Biol.

[b115] Bilodeau S, Kagey MH, Frampton GM, Rahl PB, Young RA (2009). SetDB1 contributes to repression of genes encoding developmental regulators and maintenance of ES cell state. Genes Dev.

[b116] Schotta G, Ebert A, Reuter G (2003). SU(VAR)3-9 is a conserved key function in heterochromatic gene silencing. Genetica.

[b117] Lehnertz B, Ueda Y, Derijck AA, Braunschweig U, Perez-Burgos L, Kubicek S, Chen T, Li E, Jenuwein T, Peters AH (2003). Suv39 h-mediated histone H3 lysine 9 methylation directs DNA methylation to major satellite repeats at pericentric heterochromatin. Curr Biol.

[b118] Fuks F, Hurd PJ, Deplus R, Kouzarides T (2003). The DNA methyltransferases associate with HP1 and the SUV39H1 histone methyltransferase. Nucleic Acids Res.

[b119] Fuks F, Hurd PJ, Wolf D, Nan X, Bird AP, Kouzarides T (2003). The methyl-CpG-binding protein MeCP2 links DNA methylation to histone methylation. J Biol Chem.

[b120] Hirota T, Lipp JJ, Toh BH, Peters JM (2005). Histone H3 serine 10 phosphorylation by Aurora B causes HP1 dissociation from heterochromatin. Nature.

[b121] Peters AH, Kubicek S, Mechtler K, O’Sullivan RJ, Derijck AA, Perez-Burgos L, Kohlmaier A, Opravil S, Tachibana M, Shinkai Y (2003). Partitioning and plasticity of repressive histone methylation states in mammalian chromatin. Mol Cell.

[b122] Saksouk N, Barth TK, Ziegler-Birling C, Olova N, Nowak A, Rey E, Mateos-Langerak J, Urbach S, Reik W, Torres-Padilla ME (2014). Redundant mechanisms to form silent chromatin at pericentromeric regions rely on BEND3 and DNA methylation. Mol Cell.

[b123] Puschendorf M, Terranova R, Boutsma E, Mao X, Isono K, Brykczynska U, Kolb C, Otte AP, Koseki H, Orkin SH (2008). PRC1 and Suv39h specify parental asymmetry at constitutive heterochromatin in early mouse embryos. Nat Genet.

[b124] Tardat M, Albert M, Kunzmann R, Liu Z, Kaustov L, Thierry R, Duan S, Brykczynska U, Arrowsmith CH, Peters AH (2015). Cbx2 targets PRC1 to constitutive heterochromatin in mouse zygotes in a parent-of-origin-dependent manner. Mol Cell.

[b125] Alder O, Lavial F, Helness A, Brookes E, Pinho S, Chandrashekran A, Arnaud P, Pombo A, O’Neill L, Azuara V (2010). Ring1B and Suv39h1 delineate distinct chromatin states at bivalent genes during early mouse lineage commitment. Development.

[b126] Hawkins RD, Hon GC, Lee LK, Ngo Q, Lister R, Pelizzola M, Edsall LE, Kuan S, Luu Y, Klugman S (2010). Distinct epigenomic landscapes of pluripotent and lineage-committed human cells. Cell Stem Cell.

[b127] Voigt P, LeRoy G, Drury WJ, Zee BM, Son J, Beck DB, Young NL, Garcia BA, Reinberg D (2012). Asymmetrically modified nucleosomes. Cell.

[b128] Mozzetta C, Pontis J, Fritsch L, Robin P, Portoso M, Proux C, Margueron R, Ait-Si-Ali S (2014). The histone H3 lysine 9 methyltransferases G9a and GLP regulate polycomb repressive complex 2-mediated gene silencing. Mol Cell.

[b129] Boros J, Arnoult N, Stroobant V, Collet JF, Decottignies A (2014). Polycomb repressive complex 2 and H3K27me3 cooperate with H3K9 methylation to maintain heterochromatin protein 1alpha at chromatin. Mol Cell Biol.

[b130] de la Cruz CC, Kirmizis A, Simon MD, Isono K, Koseki H, Panning B (2007). The polycomb group protein SUZ12 regulates histone H3 lysine 9 methylation and HP1 alpha distribution. Chromosome Res.

[b131] Yamamoto K, Sonoda M, Inokuchi J, Shirasawa S, Sasazuki T (2004). Polycomb group suppressor of zeste 12 links heterochromatin protein 1alpha and enhancer of zeste 2. J Biol Chem.

[b132] Rougeulle C, Chaumeil J, Sarma K, Allis CD, Reinberg D, Avner P, Heard E (2004). Differential histone H3 Lys-9 and Lys-27 methylation profiles on the X chromosome. Mol Cell Biol.

[b133] Escamilla-Del-Arenal M, da Rocha ST, Spruijt CG, Masui O, Renaud O, Smits AH, Margueron R, Vermeulen M, Heard E (2013). Cdyl, a new partner of the inactive X chromosome and potential reader of H3K27me3 and H3K9me2. Mol Cell Biol.

[b134] Wang J, Lawry ST, Cohen AL, Jia S (2014). Chromosome boundary elements and regulation of heterochromatin spreading. Cell Mol Life Sci.

[b135] Elgin SC, Reuter G (2013). Position-effect variegation, heterochromatin formation, and gene silencing in *Drosophila*. Cold Spring Harb Perspect Biol.

[b136] Narendra V, Rocha PP, An D, Raviram R, Skok JA, Mazzoni EO, Reinberg D (2015). Transcription. CTCF establishes discrete functional chromatin domains at the Hox clusters during differentiation. Science.

[b137] Jung HR, Sidoli S, Haldbo S, Sprenger RR, Schwammle V, Pasini D, Helin K, Jensen ON (2013). Precision mapping of coexisting modifications in histone H3 tails from embryonic stem cells by ETD-MS/MS. Anal Chem.

[b138] Gaydos LJ, Rechtsteiner A, Egelhofer TA, Carroll CR, Strome S (2012). Antagonism between MES-4 and Polycomb repressive complex 2 promotes appropriate gene expression in *C. elegans* germ cells. Cell Rep.

[b139] Bender LB, Suh J, Carroll CR, Fong Y, Fingerman IM, Briggs SD, Cao R, Zhang Y, Reinke V, Strome S (2006). MES-4: an autosome-associated histone methyltransferase that participates in silencing the X chromosomes in the *C. elegans* germ line. Development.

[b140] Rechtsteiner A, Ercan S, Takasaki T, Phippen TM, Egelhofer TA, Wang W, Kimura H, Lieb JD, Strome S (2010). The histone H3K36 methyltransferase MES-4 acts epigenetically to transmit the memory of germline gene expression to progeny. PLoS Genet.

[b141] Yuan G, Ma B, Yuan W, Zhang Z, Chen P, Ding X, Feng L, Shen X, Chen S, Li G (2013). Histone H2A ubiquitination inhibits the enzymatic activity of H3 lysine 36 methyltransferases. J Biol Chem.

[b142] Endoh M, Endo TA, Endoh T, Isono K, Sharif J, Ohara O, Toyoda T, Ito T, Eskeland R, Bickmore WA (2012). Histone H2A mono-ubiquitination is a crucial step to mediate PRC1-dependent repression of developmental genes to maintain ES cell identity. PLoS Genet.

[b143] Kim DH, Tang Z, Shimada M, Fierz B, Houck-Loomis B, Bar-Dagen M, Lee S, Lee SK, Muir TW, Roeder RG (2013). Histone H3K27 trimethylation inhibits H3 binding and function of SET1-like H3K4 methyltransferase complexes. Mol Cell Biol.

[b144] Cai L, Rothbart SB, Lu R, Xu B, Chen WY, Tripathy A, Rockowitz S, Zheng D, Patel DJ, Allis CD (2013). An H3K36 methylation-engaging Tudor motif of polycomb-like proteins mediates PRC2 complex targeting. Mol Cell.

[b145] Musselman CA, Avvakumov N, Watanabe R, Abraham CG, Lalonde ME, Hong Z, Allen C, Roy S, Nunez JK, Nickoloff J (2012). Molecular basis for H3K36me3 recognition by the Tudor domain of PHF1. Nat Struct Mol Biol.

[b146] Brien GL, Gambero G, O’Connell DJ, Jerman E, Turner SA, Egan CM, Dunne EJ, Jurgens MC, Wynne K, Piao L (2012). Polycomb PHF19 binds H3K36me3 and recruits PRC2 and demethylase NO66 to embryonic stem cell genes during differentiation. Nat Struct Mol Biol.

[b147] Ballare C, Lange M, Lapinaite A, Martin GM, Morey L, Pascual G, Liefke R, Simon B, Shi Y, Gozani O (2012). Phf19 links methylated Lys36 of histone H3 to regulation of Polycomb activity. Nat Struct Mol Biol.

[b148] Reynolds N, Salmon-Divon M, Dvinge H, Hynes-Allen A, Balasooriya G, Leaford D, Behrens A, Bertone P, Hendrich B (2012). NuRD-mediated deacetylation of H3K27 facilitates recruitment of Polycomb Repressive Complex 2 to direct gene repression. EMBO J.

[b149] Kim TW, Kang BH, Jang H, Kwak S, Shin J, Kim H, Lee SE, Lee SM, Lee JH, Kim JH (2015). Ctbp2 modulates NuRD-mediated deacetylation of H3K27 and facilitates PRC2-mediated H3K27me3 in active embryonic stem cell genes during exit from pluripotency. Stem Cells.

[b150] Lau PN, Cheung P (2011). Histone code pathway involving H3 S28 phosphorylation and K27 acetylation activates transcription and antagonizes polycomb silencing. Proc Natl Acad Sci USA.

[b151] Gehani SS, Agrawal-Singh S, Dietrich N, Christophersen NS, Helin K, Hansen K (2010). Polycomb group protein displacement and gene activation through MSK-dependent H3K27me3S28 phosphorylation. Mol Cell.

[b152] Pasini D, Malatesta M, Jung HR, Walfridsson J, Willer A, Olsson L, Skotte J, Wutz A, Porse B, Jensen ON (2010). Characterization of an antagonistic switch between histone H3 lysine 27 methylation and acetylation in the transcriptional regulation of Polycomb group target genes. Nucleic Acids Res.

[b153] Turkmen S, Gillessen-Kaesbach G, Meinecke P, Albrecht B, Neumann LM, Hesse V, Palanduz S, Balg S, Majewski F, Fuchs S (2003). Mutations in NSD1 are responsible for Sotos syndrome, but are not a frequent finding in other overgrowth phenotypes. Eur J Hum Genet.

[b154] Rio M, Clech L, Amiel J, Faivre L, Lyonnet S, Le Merrer M, Odent S, Lacombe D, Edery P, Brauner R (2003). Spectrum of NSD1 mutations in Sotos and Weaver syndromes. J Med Genet.

[b155] Douglas J, Coleman K, Tatton-Brown K, Hughes HE, Temple IK, Cole TR, Rahman N, Childhood Overgrowth C (2005). Evaluation of NSD2 and NSD3 in overgrowth syndromes. Eur J Hum Genet.

[b156] Morishita M, di Luccio E (2011). Cancers and the NSD family of histone lysine methyltransferases. Biochim Biophys Acta.

[b157] Kanu N, Gronroos E, Martinez P, Burrell RA, Yi Goh X, Bartkova J, Maya-Mendoza A, Mistrik M, Rowan AJ, Patel H (2015). SETD2 loss-of-function promotes renal cancer branched evolution through replication stress and impaired DNA repair. Oncogene.

[b158] Hock H (2012). A complex Polycomb issue: the two faces of EZH2 in cancer. Genes Dev.

[b159] Chan KM, Fang D, Gan H, Hashizume R, Yu C, Schroeder M, Gupta N, Mueller S, James CD, Jenkins R (2013). The histone H3.3K27M mutation in pediatric glioma reprograms H3K27 methylation and gene expression. Genes Dev.

[b160] Garcia BA, Thomas CE, Kelleher NL, Mizzen CA (2008). Tissue-specific expression and post-translational modification of histone H3 variants. J Proteome Res.

[b161] Wirbelauer C, Bell O, Schubeler D (2005). Variant histone H3.3 is deposited at sites of nucleosomal displacement throughout transcribed genes while active histone modifications show a promoter-proximal bias. Genes Dev.

[b162] Raisner RM, Hartley PD, Meneghini MD, Bao MZ, Liu CL, Schreiber SL, Rando OJ, Madhani HD (2005). Histone variant H2A.Z marks the 5′ ends of both active and inactive genes in euchromatin. Cell.

[b163] Wen H, Li Y, Xi Y, Jiang S, Stratton S, Peng D, Tanaka K, Ren Y, Xia Z, Wu J (2014). ZMYND11 links histone H3.3K36me3 to transcription elongation and tumour suppression. Nature.

[b164] Jacob Y, Bergamin E, Donoghue MT, Mongeon V, LeBlanc C, Voigt P, Underwood CJ, Brunzelle JS, Michaels SD, Reinberg D (2014). Selective methylation of histone H3 variant H3.1 regulates heterochromatin replication. Science.

